# Three-stage biochemical selection: cloning of prototype class IIS/IIC/IIG restriction endonuclease-methyltransferase TsoI from the thermophile *Thermus scotoductus*

**DOI:** 10.1186/1471-2199-14-17

**Published:** 2013-08-06

**Authors:** Piotr M Skowron, Jolanta Vitkute, Danute Ramanauskaite, Goda Mitkaite, Joanna Jezewska-Frackowiak, Joanna Zebrowska, Agnieszka Zylicz-Stachula, Arvydas Lubys

**Affiliations:** 1 Division of Molecular Biotechnology, Department of Chemistry, Institute for Environmental and Human Health Protection, University of Gdansk, Wita Stwosza 63, 80-952, Gdansk, Poland; 2 Thermo Fisher Scientific, V.A. Graiciuno 8, LT-02241, Vilnius, Lithuania; 3 Department of Botany and Genetics, Vilnius University, M.K. Ciurlionio 21/27, LT-03101, Vilnius, Lithuania

## Abstract

**Background:**

In continuing our research into the new family of bifunctional restriction endonucleases (REases), we describe the cloning of the *tsoIRM* gene. Currently, the family includes six thermostable enzymes: TaqII, Tth111II, TthHB27I, TspGWI, TspDTI, TsoI, isolated from various *Thermus* sp. and two thermolabile enzymes: RpaI and CchII, isolated from mesophilic bacteria *Rhodopseudomonas palustris* and *Chlorobium chlorochromatii*, respectively. The enzymes have several properties in common. They are large proteins (molecular size app. 120 kDa), coded by fused genes, with the REase and methyltransferase (MTase) in a single polypeptide, where both activities are affected by S-adenosylmethionine (SAM). They recognize similar asymmetric cognate sites and cleave at a distance of 11/9 nt from the recognition site. Thus far, we have cloned and characterised TaqII, Tth111II, TthHB27I, TspGWI and TspDTI.

**Results:**

TsoI REase, which originate from thermophilic *Thermus scotoductus* RFL4 (*T. scotoductus*), was cloned in *Escherichia coli* (*E. coli*) using two rounds of biochemical selection of the *T. scotoductus* genomic library for the TsoI methylation phenotype. DNA sequencing of restriction-resistant clones revealed the common open reading frame (ORF) of 3348 bp, coding for a large polypeptide of 1116 aminoacid (aa) residues, which exhibited a high level of similarity to Tth111II (50% identity, 60% similarity). The ORF was PCR-amplified, subcloned into a pET21 derivative under the control of a T7 promoter and was subjected to the third round of biochemical selection in order to isolate error-free clones. Induction experiments resulted in synthesis of an app. 125 kDa protein, exhibiting TsoI-specific DNA cleavage. Also, the wild-type (wt) protein was purified and reaction optima were determined.

**Conclusions:**

Previously we identified and cloned the *Thermus* family RM genes using a specially developed method based on partial proteolysis of thermostable REases. In the case of TsoI the classic biochemical selection method was successful, probably because of the substantially lower optimal reaction temperature of TsoI (app. 10-15°C). That allowed for sufficient MTase activity *in vivo* in recombinant *E. coli*. Interestingly, TsoI originates from bacteria with a high optimum growth temperature of 67°C, which indicates that not all bacterial enzymes match an organism’s thermophilic nature, and yet remain functional cell components. Besides basic research advances, the cloning and characterisation of the new prototype REase from the *Thermus* sp. family enzymes is also of practical importance in gene manipulation technology, as it extends the range of available DNA cleavage specificities.

## Background

Subtype IIS REases, unlike classic Type II REases which recognise palindromic DNA sequences and cleave within those sites, bind an asymmetric DNA sequence and cleave outside it at a defined distance, up to 21 nt, regardless of the sequence within the cleavage site [1,2]. Further structural and functional complications include atypical IIS enzymes, which are a fusion of REase and MTase in a single polypeptide (Subtype IIC) and/or require/are stimulated by SAM (Subtype IIG). Further diversity within Subtypes IIS/IIC/IIG [3-8] is a family of enzymes grouping REases originally found in *Thermus* sp., which includes TsoI. Since we are aiming at studying the *Thermus* sp. IIS/IIC/IIG enzymes family, we have undertaken cloning, expression and characterisation of TsoI. The *Thermus* sp. family enzymes recognise 5–6 bp DNA sequences which show certain similarities: TspGWI [5′-ACGGA-3’ (11/9) [3], TaqII [(5’-GACCGA-3’ (11/9) [8] or 5’-GACCGA-3’ and 5’-CACCCA-3’ (11/9) [7,9], TspDTI [(5’-ATGAA-3’ (11/9) [4]], Tth111II/TthHB27I isoschizomers [(5’-CAARCA-3’ (11/9) [2,8,10]] and TsoI [5’-TARCCA-3’ (11/9) [2,8], (this work)]]. As detected by bioinformatics analysis, enzymes from mesophilic bacteria – RpaI, recognising the 7-bp degenerate sequence 5’-GTYGGAG-3’ (11/9) and CchII, recognizing 5’-GGARGA-3’ (11/9) apparently belong to the *Thermus* sp. family as well [2,11]. The family shares common biochemical features (summarized in Table one in reference [2]), such as a large molecular size of approximately 120 kDa, REase activity affected by SAM or its analogues, similarities in amino acid (aa) sequences despite distinct specificities, an identical cleavage distance of 11/9 nt downstream from the recognition site and the domain architecture related to simplified Type I REases. All characterised family members originate from the genus *Thermus*, suggesting that they have evolved from a common ancestor [4]. Further comparison has revealed that the group is further internally diversified [6,8]. Bioinformatics analysis and site-directed mutagenesis have led to the differentiation between two subfamilies of closely related enzymes: the TspDTI-subfamily, containing TspDTI, Tth111II/TthHB27I, TsoI, CchII and the TspGWI-subfamily, which includes TspGWI, TaqII and RpaI [2,3,6,9]. Besides aa sequence homologies, the subfamilies are also differentiated by the types of their catalytic motifs: the TspDTI-subfamily has atypical REase catalytic motif D-EXE (also detected previously in typical Type II BamHI REase [12]) and cysteine+serine containing SAM binding motif (D/P)PACGSG, while the TspGWI-subfamily has typical REase catalytic motif PD-(D/E)XK and SAM binding motif (DPA(V/M)GTG [6,8]. Moreover, the TspGWI-subfamily exhibits an interesting feature: a novel phenomenon of REase specificity change, induced by a cofactor analogue. Both TspGWI and TaqII can be converted to very frequent app. 3 bp cutters from canonical 5–6 bp sites by replacing SAM with its analogue sinefungin (SIN), with reversed charge distribution ([5,13]; in press). These chemically-induced changes in the recognition sequence differ from the well-known “star activity” phenomenon. They are apparently a result of SIN interaction with an allosteric pocket on the protein surface which binds the stimulatory SAM molecule. Hence, two more novel prototype specificities have been generated by chemical means [5]. Because of the very high frequency of DNA cleavage, they are uniquely suited for use as molecular tools for generating quasi-random genomic libraries [14].

## Results and discussion

### Cloning, sequencing and analysis of the *tsoIRM* gene

**Figure 1:**
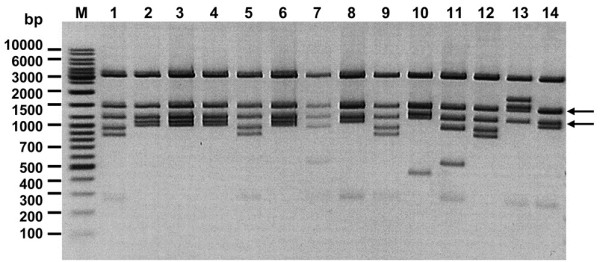
**Restriction analysis of large recombinant plasmids isolated during the biochemical selection of *T. scotoductus *library.** Patterns of DNA fragments resulting from the HindIII cleavage of recombinant plasmids, isolated after two rounds of biochemical selection using TsoI, visualized on electrophoretic agarose/Tris-borate-EDTA (TBE) gel (1%), stained with ethidium bromide (EtBr). Arrows show cloned fragments of 1.75 and 2 kb in size, which originate from *T. scotoductus* and are common for all TsoI-selected plasmids examined. Lane M - DNA standards (Gene Ruler™ Ladder Mix (Thermo Fisher Scientific (Fermentas); selected bands marked).

Following our studies of the *Thermus* sp. family of enzymes [4], we cloned the genes coding for TspGWI [6], TspDTI [8], TaqII [GenBank: AY057443, AAL23675; manuscript submitted], TthHB27I/Tth111II [manuscript in preparation] and TsoI [this work; GenBank: KC503938]. Previously, as a result of the enzymes’ feature of incomplete DNA digestion, complicating the application of known methods of biochemical selection both for the methylation phenotype [15] or the related ‘white-blue’ screen for DNA damage/modification [16-18], we developed an *in vitro* approach for thermophilic REase cloning [6,8]. TsoI has a substantially lower reaction optimum than other *Thermus* sp. REases - app. 10-15°C. Thus, according to the chemical rule whereby reaction speeds decrease 2–3 fold per 10°C drop in reaction temperature, it was assumed that TsoI would retain a substantially higher activity *in vivo* in recombinant *E. coli* cells at 37°C than other *Thermus* sp. REases. Accordingly, we expected specific methylation *in vivo* of plasmids carrying the cloned TsoI MTase gene at a high enough level to allow the classic biochemical selection method to be used [15]. Due to the partial cleavage feature of TsoI, however, selection difficulties were anticipated. Thus, a new variant of the classic method of selecting for the methylation phenotype of recombinant “positive” clones was developed. An integral part of the procedure was the use of the proprietary positive selection vector pSEKm’-MCS (Thermo Fisher Scientific/Fermentas). The vector features resistance for both ampicillin and kanamycin, possesses five TsoI targets and selects for any cloned insert, thus decreasing the cloning background which may have resulted from self-ligated vector molecules. Non-specific selection of recombinant plasmids was combined with two rounds of the classic biochemical selection method for the TsoI methylation phenotype. Since the vector included five target recognition sites for TsoI, it was expected to be an efficient substrate for TsoI REase. Notwithstanding, transformation yielded 8 × 10^5^ colonies, thus providing an over 1000-fold genome coverage. Such a high coverage seems preferable in order to increase the chances of obtaining intact REase genes, as when cloning highly toxic genes, such as those coding for REases. Owing to the negative selection pressure in the recombinant *E. coli* host against detrimental plasmids, libraries tend to be non-representative. Considering the partial digestion feature of TsoI and the lower enzyme activity *in vivo* at 37°C, a departure from the standard biochemical selection procedure was made: following plasmid DNA isolation from colonies collected as a pool and digestion with excess TsoI, re-transformation and repeated plating, no analysis of the surviving colonies was performed. Instead, the next round of pooled *in toto* colonies from the first round of selection was subjected to repeated plasmid DNA isolation, excess TsoI cleavage, re-transformation and re-plating. After two biochemical selection rounds, 50 clones were analysed by colony PCR to screen for plasmids carrying fragments larger than 3 kb (the expected size of the *tsoIRM* gene). Figure 1 shows that 14 HindIII-digested large recombinant plasmids isolated during the screening procedure which share common cloned fragments of 1.2 and 1.5 kb in size, suggesting that all contain the same genetic locus from *T. scotoductus*.

**Figure 2:**
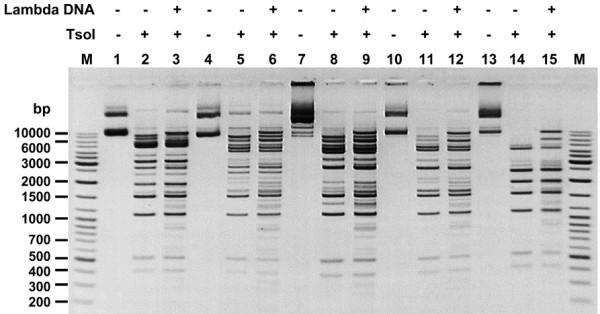
**Evaluation of resistance to TsoI digestion of plasmids, obtained after two rounds of biochemical selection.** Lanes M – DNA standards (Gene Ruler™ Ladder Mix (Thermo Fisher Scientific (Fermentas); selected bands marked); lanes 1, 4, 7, 10 and 13 - DNAs of individual plasmids, untreated; lanes 2, 5, 8, 11 and 14 - DNAs of individual plasmids treated with TsoI; lanes 3, 6, 9, 12 and 15 - DNAs of individual plasmids supplemented with λ DNA and treated with TsoI. Samples were resolved on 1.2% agarose gel in TBE buffer, stained with EtBr.

**Figure 3:**
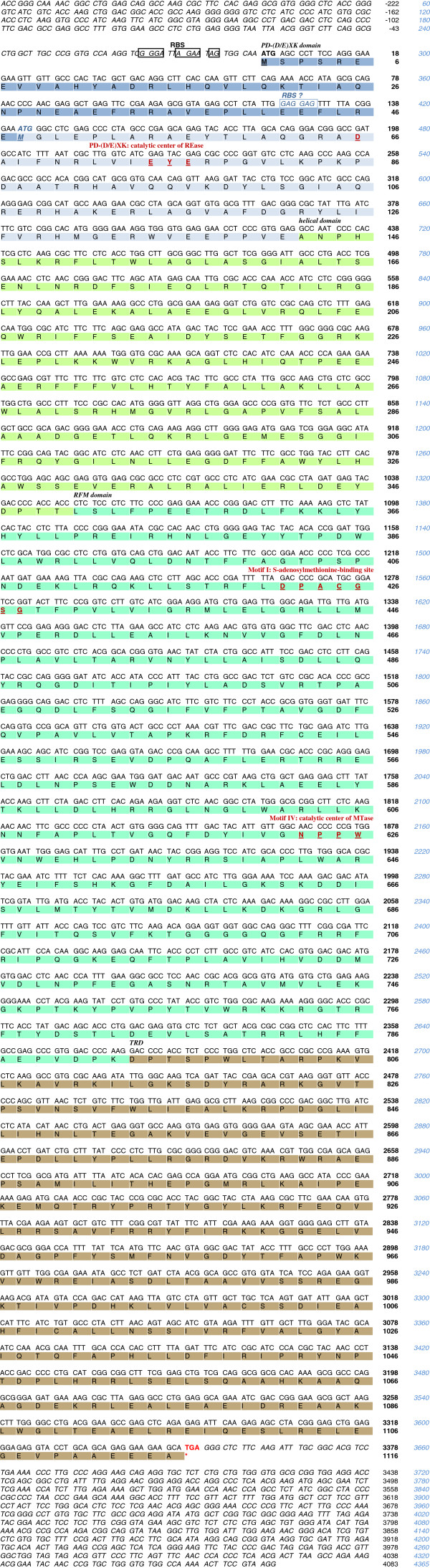
**DNA, amino acid sequence and functional motifs of the *tsoIRM *gene and its flanking regions and schematic organization of the enzyme domains.** The predicted amino acid sequence of the 127.6 kDa TsoI protein is indicated in capital letters. The DNA sequence is numbered in two styles: (*i*) black numbering starts with negative values, with +1 nt marking the beginning of the *tsoIRM* ORF, (*ii*) the blue numbering corresponds to the entire DNA sequence deposited in the GenBank under acc. number KC503938] (includes *tsoIRM* gene and flanking regions). The ATG START codon is in black bold. The TGA stop codon is shown in red. The potential *tsoIRM* ORF Ribosome Binding Sites (RBS) are boxed. The potential internal ATG START is in blue. The potential *tsoIRM* internal ORF RBS is boxed in blue. The crucial amino acids of the catalytic centres are dark red, bold and underlined. The functional protein domains are marked as follows: REase domain in blue, helical domain in light green, MTase domain in dark green, TRD in brown.

The initial verification of clones, based on the detection of common restriction fragments (Figure 1), was followed by functional analysis. The putative *tsoIRM* gene was expressed at a detectable level in *E. coli*, since its presence was high enough to ensure protection against TsoI digestion in the biochemical selection procedure. DNA of five individual recombinant plasmids were digested in excess TsoI and analysed using agarose gel electrophoresis (Figure 2). As shown in Figure 2, only traces of completely protected plasmid DNA were observed. Inclusion of λ DNA into parallel reactions served as an internal control which revealed that (*i*) DNA of isolated plasmids do not interfere with the activity of TsoI, and (*ii*) the same minute amount of completely protected plasmid DNA was observed in both reactions with/without λ DNA, which suggest that the TsoI-specific methylation was probable cause of plasmid DNA resistance to cleavage. These *in vivo* results were subjected to further validation by *in vitro* assays for the native TsoI methylation activity using homogeneous enzyme. Under a variety of conditions tested, the MTase specific activity was substantially lower than that of the REase (not shown). Thus, the MTase activity *in vivo* was probably enhanced by cytoplasmic environment and/or extended incubation of cells prior to plasmid isolations. This has provided enough reaction time to significantly protect DNA from further TsoI REase cleavage. Taken together, selection difficulties were possibly related to both the partial cleavage feature of TsoI and incomplete DNA protection by TsoI MTase activity. What’s worthy of note, a relatively low level of protection explains why two rounds of biochemical selection were required in order to enrich the library by plasmids carrying the cloned *tsoIRM* gene. The clones shown in Figure 2 were subjected to sequencing of inserted fragments, first by using vector-specific primers and then by insert-specific primers. As a result, the combined and cross-checked 4365-bp long genomic contig from *T. scotoductus* RFL4 was determined [GenBank: KC503938]. The fragment, as analysed by DNASIS MAX software, contained 3348 bp ORF, which encoded a putative long polypeptide, exhibiting a high level of similarity with the Tth111II bifunctional REase-MTase (50% identity, 60% similarity). Since the ORF sequence-based predicted molecular weight of putative 1116 aa TsoI is 127.6 kDa (DNASIS MAX and Vector NTI calculations) which matches very well our previously published SDS/PAGE results of native TsoI (app. 120 kDa) [8], we concluded that the detected cloned ORF indeed codes for the *tsoIRM* gene. Further bioinformatics analysis has revealed that TsoI is moderately basic, with a calculated pI of 8.06-8.11 (Vector NTI and DNASIS MAX calculations, respectively) (the only basic member of the *Thermus* sp. enzyme family). No sequence similarity of TsoI to any MTase or DNA-binding protein was found in the flanking regions of the ORF. There is only a single TsoI recognition site present within the ORF. The ORF begins with the ATG START codon and contains 3 putative upstream RBSs: - 7 AG, -10 AGAA and -16 GGGA. Note therefore, that within the ORF there is a second potential ATG START codon, located at residue 48, with a ribosome-binding site upstream GAGGAG, located at a sub-optimal distance of −12 (Figure 3). Translation from the second start codon would result in slightly smaller protein of 1069 aa and 122.1 kDa. The ORF of 1116 aa is GC rich (56.54%); nevertheless, it is markedly lower than other *Thermus* sp. family coding genes, except *tspDTIRM*[8]. Thus, like *tspDTIRM*, *tsoIRM* may have been acquired/evolved differently than other *Thermus* sp. genes, which may have included horizontal gene transfer from a lower GC content bacteria. According to the previously published bioinformatics analysis [8], TsoI exhibited similarity to several known and putative Type IIC/IIG enzymes, including the previously characterised nucleases TthHB27I/Tth111II isoschizomer pair and TspDTI with alignment covering essentially the whole length of the polypeptide. Despite such a high sequence similarity between TthHB27I/Tth111II and TspDTI enzymes, TsoI has a different sequence specificity – 5’-TARCCA-3’ [2,8] to TspDTI (5’-ATGAA-3’) and TthHB27I/Tth111II (5’-CAARCA-3’) – while cleaving at the same distance of 11/9 nt from the recognition site. Nevertheless, all of these asymmetric cognate sequences share two common adenine residues, located at the same positions. In contrast, two other Type IIC/IIG enzymes from *Thermus*, i.e. TspGWI [GenBank: EF095488, ABO26710] and TaqII [GenBank: AY057443, AAL23675], showed very low sequence similarity between TspDTI, TthHB27I/Tth111II and TsoI in pairwise comparisons, dividing the *Thermus* sp. enzymes family into two sub-families. Nevertheless, both sub-families share a common organisation scheme, sharing a modular structure with the same linearly located functional/physical domains of very similar sizes [8]. This scheme is followed by TsoI, which has consecutive fused segments, starting from the N-terminus: (*i*) DNA cleavage/Mg^2+^ -binding (including the atypical D-EXE motif, approximate aa 1–160), (*ii*) a helical region/interaction between domains (fusion subunits link and potentially regulatory region, app. aa 160–360) - recently, the crystal structure of a Type IIC/IIG bifunctional BpuSI REase was established and an alpha-helical domain connecting the REase and MTase domains was suggested to link and regulate structure as well as domain communication - furthermore, it may determine the cleavage distance from the recognition site [19], (*iii*) DNA m6A methylation (including SAM binding motif PPACGSG and methylation catalytic motif NPPW, app. aa 360–790) and (*iv*) DNA sequence recognition region (possibly including two Target Recognition Domains (TRDs), app. aa 790-C-terminus) [8] (Figure 3). Overall, this organisation resembles the simplified (fused in the same polypeptide) HsdR, HsdM and HsdS subunit domain architecture of Type I REases [8].

### Expression analysis of the cloned ***tsoIRM*** gene

**Figure 4:**
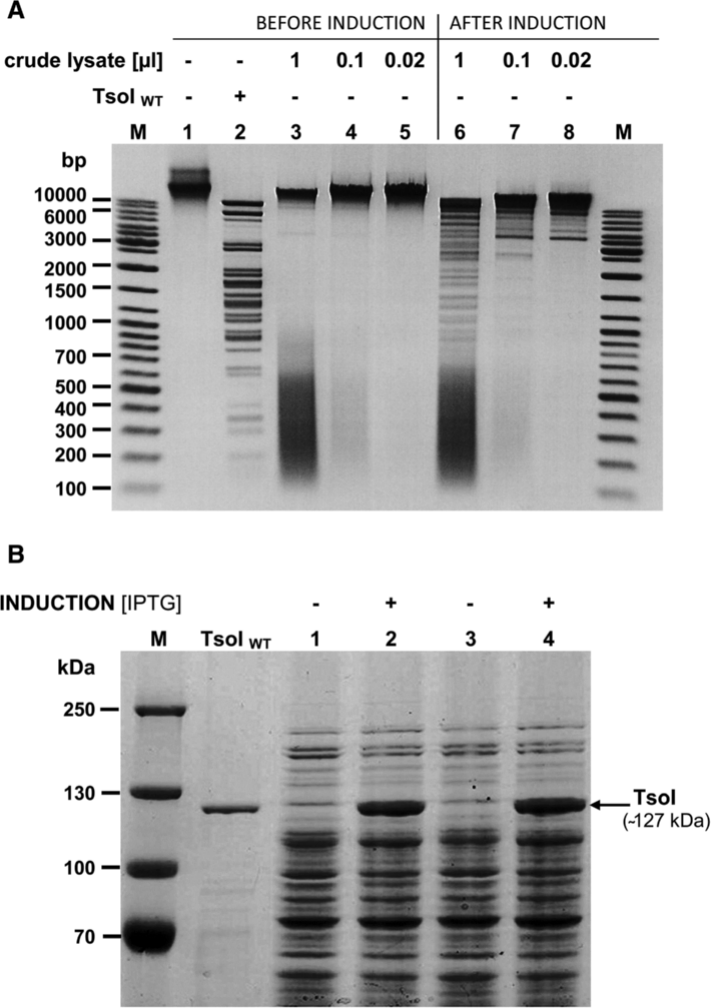
**Analysis of the induction pattern of recombinant TsoI REase. (A)** Enzymatic activity in crude *E. coli* ER2566/pET21NS-*TsoIRM* extracts. Two selected clones of the confirmed *tsoIRM* gene nucleotide sequence after the 3^rd^ round of biochemical selection were subjected to expression experiments, giving similar TsoI induction patterns. For clarity, the expression experiment of only one selected clone was shown. Lanes M, DNA size standards (Gene Ruler^TM^ Ladder Mix (Thermo Fisher Scientific (Fermentas); selected bands marked); lane 1, control (untreated) λ DNA; lane 2, λ DNA cleaved with native (wt) TsoI; lanes 3, 4, 5, various amounts (1, 0.1, 0.02 μl, respectively) of cell extracts prior to induction incubated with λ DNA in TsoI digestion buffer; lanes 6, 7, 8, various amounts (1, 0.1, 0.02 μl, respectively) of induced cell extracts incubated with λ DNA in TsoI digestion buffer. Samples were resolved on 1.2% agarose gel in TBE buffer, stained with EtBr. **(B)** SDS/PAGE evaluation of TsoI induction in *E. coli* ER2566/pET21NS-*TsoIRM*. Two selected clones as in A were analysed for the presence of TsoI protein bands on 8% SDS/PAGE gels. Electrophoresis was subjected to an extended run in order to clearly visualize protein bands in the high molecular weight range. Lanes M, protein standards (Gene Ruler^TM^ Prestained Protein Ladder Plus (Thermo Fisher Scientific (Fermentas); lane TsoI_WT_, native (wt) TsoI (1 unit); lanes 1 and 3, samples prepared from two colonies of *E. coli* ER2566/pET21NS-*TsoIRM* clones as in A, before induction; lanes 2 and 4, as in lanes 1 and 3, after IPTG induction.

**Figure 5:**
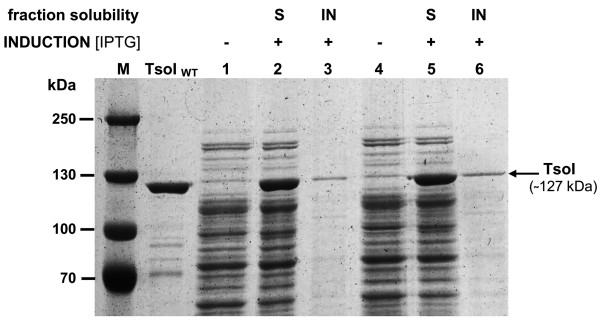
**Analysis of the solubility of expressed recombinant TsoI REase SDS/PAGE (8%) analysis of induced versus uninduced and soluble versus insoluble fractions.** Lane M, protein standard (PageRuler™ Prestained Protein Ladder Plus (Thermo Fisher Scientific (Fermentas); lane TsoIWT, wt TsoI (2 units); lanes 1 and 4, samples prepared directly from two *E. coli* ER2566 colonies resulting from transformation with the same pET21NS-*TsoIRM* plasmids, before induction; lanes 2 and 5, the same as in lanes 1 and 3, but after IPTG induction (soluble fraction); lanes 3 and 6, the same as in lanes 2 and 5 (insoluble fraction).

**Figure 6:**
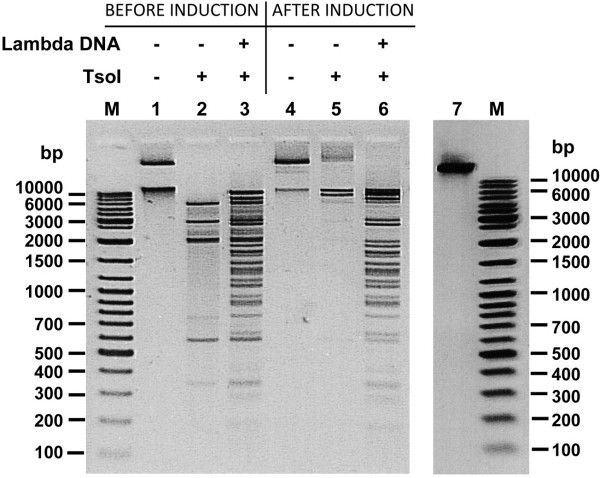
**Evaluation of the TsoI-resistance of plasmids isolated from the same culture before and after TsoI synthesis induction.** Lane M, DNA standard (Gene Ruler™ Ladder Mix (Thermo Fisher Scientific (Fermentas); selected bands marked); lanes 1 and 4, uncleaved pET21NS-*TsoIRM* plasmid DNA; lanes 2 and 5, pET21NS-*TsoIRM* after incubation with TsoI; lanes 3 and 6, pET21NS-*TsoIRM* supplemented with λ DNA and then incubated with TsoI.

Since the primary clones expressed the TsoI REase-MTase at a very low level, as judged by recombinant plasmid partial protection *in vivo*, further constructs were made by subcloning of PCR-amplified *tsoIRM* gene into a pET-derivative vector pET21NS. The minor Multiple Cloning Site (MCS) modification (not shown) was made to allow for directional cloning of the NotI-SmiI cleaved PCR fragment encompassing the full-length TsoI-coding gene. The TsoI ORF was placed under the control of the T7 promoter and strong RBS. IPTG-induction was used to evaluate whether the cloned *tsoIRM* gene was indeed expressed. Crude cell extracts from small scale induced cultures of individual clones revealed a trace amounts of TsoI REase activity, while SDS-PAGE demonstrated abundant amounts of a large enzyme (app. 120 kDa) that appeared after induction (Figures 4,5). Testing of the TsoI protection level of 15 individual plasmids, chosen from PCR subcloning, revealed that they are all unprotected before induction and only partially protected after induction, suggesting that either the chosen PCR conditions may have been favourable for the appearance of errors, or there may have been selective pressure for growth of only those colonies which contain plasmids coding for mutants of TsoI with reduced either the REase, MTase or both activities. Sequencing results of a few of these clones revealed the presence of multiple mutations, which most probably appeared during PCR and were further spontaneously selected *in vivo*, due to lower toxicity to *E. coli* host. In order to isolate clones, which encode highly active TsoI, 5000 ampicillin-resistant colonies obtained after the transformation of expression host *E. coli* ER5266 with a ligation mixture of expression vector and PCR amplified *tsoIRM* gene were subjected to a third round of biochemical selection. Transformants were pooled without separate cultivation of single clones and used directly to inoculate 100 ml LB media (supplemented with ampicillin). Cells were grown at 37°C until the mid-log phase, the T7 promoter was induced by the addition of IPTG, the culture was grown further at 37°C for 4 hours and then used for isolating total plasmid DNA. The latter was cleaved with TsoI and the reaction mixture was introduced back to *E. coli* ER2566. The 20 resulting colonies were again tested for TsoI activity in crude cell extracts and for protection level against TsoI cleavage of recombinant plasmids isolated from induced cultures. In contrast to previous experiments, the crude cell extracts in this case exhibited a much higher TsoI REase activity (Figure 4A), while plasmids, containing the cloned *tsoIRM* gene and isolated from induced strains, were almost completely protected from TsoI cleavage, indicating an adequate MTase activity at the same time (Figure 6, lanes 4, 5 and 6). Four of the selected plasmids were subjected to insert sequencing, and two of them were found to have no mutations in the PCR-amplified TsoI-coding gene, while expressing large amounts of TsoI protein (Figure 4B). One of these plasmids (pET21NS-TsoIRM) was used for further protein isolation and characterisation experiments.

### Characterisation of TsoI protein

**Figure 7:**
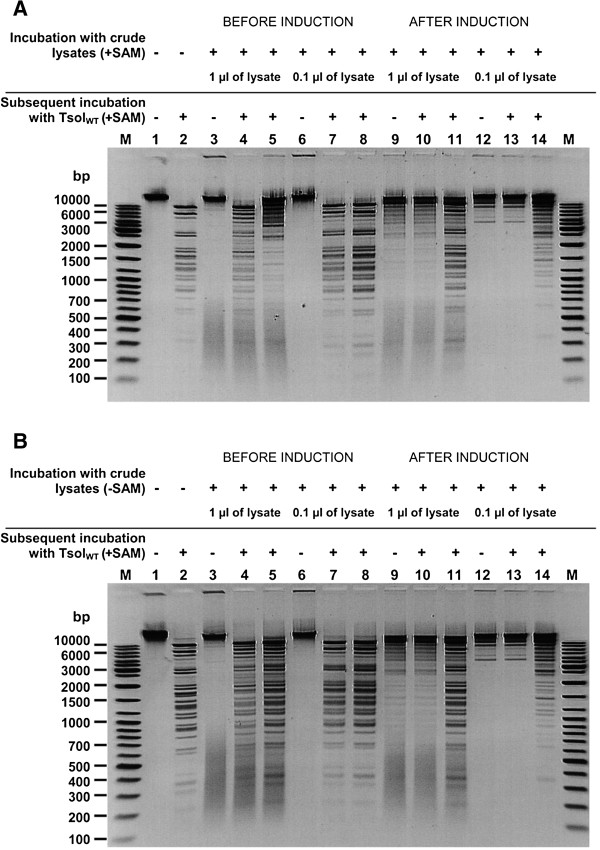
**Evaluation of TsoI REase/MTase activities in cleared lysates. (A)** Evaluation in the presence of SAM. Lanes M, DNA standard (Gene Ruler™ Ladder Mix (Thermo Fisher Scientific (Fermentas); selected bands marked); lane 1, λ DNA; lane 2, λ DNA, cleaved with wt TsoI; lanes 3, 6, 9, 12, λ DNA after incubation with indicated amounts of cleared lysates prepared from cultures: (*i*) before TsoI induction (3, 6) or (*ii*) after induction (9, 12) in the standard reaction buffer supplemented with SAM; lanes 4, 7, 10, 13, the same as in lanes 3, 6, 9, 12, but after chloroform extraction, isopropanol precipitation and subsequent incubation of dissolved samples with wt TsoI; lanes 5, 8, 11, 14, the same as in lanes 4, 7, 10, 13, except that λ DNA was added before incubation with wt TsoI in order to assess the putative inhibitory effect of the chloroform-extracted DNA on wt TsoI. **(B)** Evaluation in the absence of SAM. Description of lanes as in **A**, with the exception that SAM was not included into the reaction mixtures used to evaluate activities of crude lysates.

**Figure 8:**
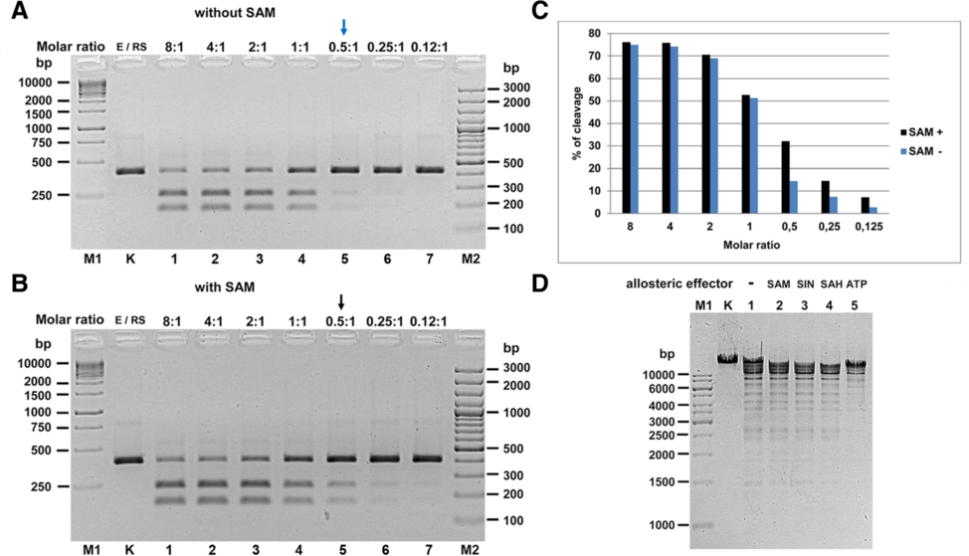
**Evaluation of the effect of cofactor SAM and its analogues on TsoI activity.** 0.3 μg (= 0.6 pmol recognition sites) single site PCR substrate (390 bp) was digested with decreasing amounts of wt TsoI for 16 h at 55°C in the standard reaction buffer: 10 mM Tris–HCl, pH 7.5, 10 mM NaCl, 10 mM MgCl_2_, 0.01 mg/ml BSA, 0.5 mM DTTn the presence or absence of SAM. **(A)** DNA cleavage in the absence of SAM. Lane M1, GeneRuler™ 1 kb DNA Ladder (Thermo Fisher Scientific (Fermentas); selected bands marked); lane K, undigested PCR fragment; lanes 1–7, digested PCR fragment: lane 1, with 4.8 pmol; lane 2, with 2.4 pmol; lane 3, with 1.2 pmol; lane 4, with 0.6 pmol; lane 5, with 0.3 pmol; lane 6, with 0.15 pmol; lane 7, with 0.075 pmol; lane M2, GeneRuler™ 100 bp Plus DNA Ladder (Thermo Fisher Scientific (Fermentas); selected bands marked). **(B)** DNA cleavage in the presence of 50 μM SAM. Lane M1, GeneRuler™ 1 kb DNA Ladder (Thermo Fisher Scientific (Fermentas); selected bands marked); lane K, undigested PCR fragment; Lanes 1–7, samples were digested with decreasing amounts of TsoI as described in **(A)**; lane M2, GeneRuler™ 100 bp Plus DNA Ladder (Thermo Fisher Scientific (Fermentas); selected bands marked). **(C)** The influence of enzyme to recognition site ratio on TsoI DNA cleavage in the presence or absence of SAM. **(D)** Effect of allosteric cofactors on wt TsoI REase activity. Three putative cofactors or analogues (SAM, SAH, SIN) as well as ATP were compared for their influence on TsoI DNA digestion activity. 0.5 μg of λ DNA (= 0.016 pmol recognition sites) was digested with 0.7 pmol (0.048 u) of TsoI in standard TsoI buffer supplemented with 50 μM of the appropriate effector and 0.5 mM DTT for 30 min at 55°C. Lane M, GeneRuler™ 1 kb DNA Ladder (Thermo Fisher Scientific (Fermentas); selected bands marked); lane K, untreated λ DNA; lane 1, λ DNA cleaved with wt TsoI (no cofactors, except Mg^2+^); lane 2, (+TsoI wt, +SAM); lane 3, (+TsoI wt, +SIN); lane 4, (+TsoI wt, +SAH); lane 5, (+TsoI wt, +ATP). DNA was treated with low amount of TsoI to pinpoint differences in the stimulatory effect. The reaction products were resolved on 1.2% agarose gel in TBE buffer and stained with EtBr.

As shown in the previous section, the expression experiments resulted in the appearance of specific DNA cleavage activity as well as large amounts of a high molecular weight recombinant polypeptide band of app. 120 kDa, which was in fact the dominant protein band in the recombinant *E. coli* lysate. The observed protein location on the SDS/PAGE gel is in very good agreement with the predicted protein size of 127.6 kDa coded by the *tsoIRM* gene. The band on SDS/PAGE, corresponding to the recombinant protein, also perfectly matches the position on the gel of purified wt TsoI from *T. scotoductus* (Figure 4B, lane TsoIWT). On the other hand, preliminary estimation of the TsoI activity in crude cell extract allowed the conclusion to be drawn that it is much lower than it could be expected based on the amount of enzyme synthesised, and suggested that either the recombinant enzyme is insoluble or has lower specific activity. In order to identify the cause for the discrepancy between TsoI activity and its intracellular amount, solubility studies of recombinant TsoI were conducted. The results in Figure 5 clearly indicate that TsoI, when expressed in *E. coli*, apparently retains a soluble conformation. Therefore, disproportionally low endonucleolytic activity of the induced TsoI may be either due to the slower turnover of recombinant enzyme compared with the wt isolate, or higher MTase activity, which (theoretically) could dominate and modify substrate DNA, preventing it from undergoing TsoI endonucleolytic cleavage. If so, dominant MTase activity could explain the apparently lower REase activity of recombinant TsoI. In order to test this idea, bacteriophage λ DNA was incubated in the presence of SAM and Mg^2+^ with cleared lysates prepared from induced and uninduced cultures (Figure 7A). The use of cleared lysates was not problematic, owing to the relatively high concentration of expressed recombinant TsoI. Subsequently, reaction products were purified by chloroform extraction and isopropanol precipitation, following dissolution in TsoI reaction buffer (supplemented with SAM), which were incubated with wt TsoI. The experiment clearly demonstrated that the induced culture not only has quite a weak endonucleolytic activity (Figure 7A, lanes 9 and 12), but also a substantial TsoI MTase activity, which is manifested by the conversion of bacteriophage λ DNA into a partially cleaved yet completely modified form that is resistant to cleavage by the subsequent addition of wt (*T. scotoductus* – isolated) TsoI (Figure 7A, lanes 10 and 13). The additional amount of unmodified λ DNA was subsequently cleaved by adding wt TsoI either completely (Figure 7A, lanes 8 and 11) or partially (lanes 5 and 14), thus suggesting that chloroform extracted and isopropanol precipitated substrates had inhibitory effect on wt TsoI in some cases. Taken together, conclusion could be made that the substrate was completely methylated during incubation with cleared lysate of induced culture in the presence of SAM (Figure 7A). Furthermore, methylation appears to be complete even when a very small amount of cleared lysate is used (Figure 7A, lane 13). These results were compared with further assays using purified, homogeneous wt TsoI, which in turn failed to show that the specific activity of TsoI MTase was higher than that of TsoI REase, regardless of the variety of conditions tested (not shown). However, the standard MTase assay, based on *in vitro* protection against a subsequently added cognate REase, while yielding quantitative results in the case of classic Type II REases, is not perfect for analysis of Subtype IIG/IIC REases. In the case of TsoI, the assay was complicated due to at least three factors: (*i*) fusion of both activities in the same polypeptide, thus allowing for concurrent actions, when divalent cations were present, (*ii*) the REase and MTase protein domains interactions, including SAM binding/allosteric effect and (*iii*) incomplete cleavage by TsoI REase, which prevents from precise distinguishing protected DNA from uncleaved DNA. Other reasons which may explain the difference between higher TsoI MTase activity *in vivo* and in crude lysates as compared to purified wt TsoI, may include: (*i*) protective effect of the high concentration of cellular proteins present *in vivo* and in crude lysates stabilising TsoI MTase, (*ii*) an important cytoplasmic component for methylation is missing or (*iii*) selective inactivation of the MTase domain during purification, while the TsoI REase domain remains functionally intact. This interwound REase-MTase activities’ relationship is further complicated by the fact that TsoI is a very slow enzyme. To test whether TsoI REase is a multi- or single turnover enzyme, the serial dilutions under controlled enzyme:recognition sites molar ratios were performed, both in the presence and absence of SAM (Figure 8). Reactions were performed for a prolonged time (overnight), to allow consecutive cleavage reaction cycles. Results shown in Figure 8ABD clearly show that at a molar ratio 1:1 and lower, a single TsoI REase molecule on average performs less than 1 cleavage per single cognate site. Considering that a competitive TsoI MTase reaction may not proceed in an experiment without SAM (Figure 8A), unless some tightly bound SAM is carried over during purification, the conclusion can be drawn that either TsoI REase is a single turnover enzyme or a majority of TsoI molecules were inactivated during purification, diluting functional enzyme molecules with non-active ones. Thus the turnover issue was not conclusively resolved, nevertheless it was confirmed that TsoI is a very “slow” enzyme, as even a very long reaction time did not result in substrate digestion exceeding a 1:1 molar ratio. TsoI approaching the characteristics of a single-turnover may represent an intermediate evolutionary stage.

Based on the fact that the MTase activity of TsoI is entirely dependent on SAM, whereas the activation of the REase function by SAM is weak, although noticeable both during prolonged digestions (Figure 8AB lanes 0.5:1 and Figure 8D) and time-limiting conditions (Figure 8C), the idea was proposed that the MTase activity of recombinant TsoI might use TsoI-bound (carried over) SAM which potentially could make a difference between activities of wt and recombinant TsoI variants. In order to test this idea, the same experiment as in Figure 7A was repeated, without the addition of SAM (Figure 7B). Bearing in mind the absolute prerequisite of the presence of SAM for the MTase DNA modification reaction, we expected to obtain the same result if the enzyme used bound SAM, and less methylation if this was not the case. The results shown in Figure 7B are nearly identical to those shown in Figure 7A, suggesting that either the cleared lysate of induced culture has a sufficiently high concentration of SAM which is enough to promote efficient DNA methylation even when a very small amount of the cleared lysate was used (Figure 7B, lanes 12 and 13), or that the recombinant enzyme has SAM already bound. Previously, we suggested that the *Thermus* sp. family enzymes might have two physically separate binding sites for SAM: one for allosteric stimulation of REase activity and another for typical SAM binding/methylation [8]. However, the important conclusion from these two experiments is that, regardless of the source of the necessary cofactor for the MTase reaction, the MTase activity of recombinant TsoI is predominant over REase activity under the same reaction conditions, when tested on crude lysates, which mimics reaction conditions *in vivo*. Such an REase-unfavourable equilibrium between the two activities of TsoI raises the question of how effective is DNA cleavage by TsoI *in vivo* in its natural host *T. scotoductus* and whether indeed the primary function of this bifunctional REase-MTase is defence against invading foreign DNA. It is possible that other functions, such as participation in recombination, by rare DNA cleavage, area primary goal of this system. Further characterization studies were conducted on a wt *T. scotoductus*-isolated homogeneous TsoI preparation. Initial tests on the newly found wt TsoI prototype enzyme have indicated that SAM slightly stimulates TsoI REase activity [2]; (Lubys Arvydas, personal communication). Hence, three potential effectors, adjudged from our previous work [3-8], were compared for their influence on TsoI activity: SAM, a natural and obligatory co-substrate for MTase activity and an allosteric stimulator for *Thermus* sp. REase activities; SIN, a SAM analogue, which apparently causes subtle changes in tertiary *Thermus* sp. REases structures, either stimulating DNA cleavage [6-8] and/or causing substrate specificity changes towards much more frequent cleavage [5]; (BMC Genomics, in press) and S-adenosylhomocysteines (SAH), the methylation reaction by-products. These results as well as TsoI digestions performed for a prolonged time under various enzyme: substrate molar ratios show that the activation of REase function by SAM, SIN and SAH is weak, but detectable (Figure 8). Nevertheless, this may lead to indirect conclusion that the enzyme retained some capability for allosteric interaction of the TsoI protein with SAM and its analogues, even though this interaction is not fully functional. A more precise answer to the interesting problem of the pleiotropic effect of SAM as well as the enzyme’s inability to conduct multiple cleavage reactions signalled in Figure 7 and Figure 8 would come from detailed *in vitro* studies using DNA-band-shift-assay, radiolabelled SAM and DNA as well as TsoI mutants with an inactivated REase catalytic motif and/or MTase catalytic motifs (work in progress). ATP was also tested as potential effector, even though it is chemically more distant molecule as compared to SAM. However, since the *Thermus* sp. enzymes resemble “streamlined halves” of Type I REases [6,8], the possibility of ATP effect, was evaluated. In addition, unusual Type II REase of eukaryotic origin – CviJI is stimulated by ATP while its specificity changes [14]. Nevertheless, ATP had no effect on TsoI (Figure 8, lane 5).

**Figure 9:**
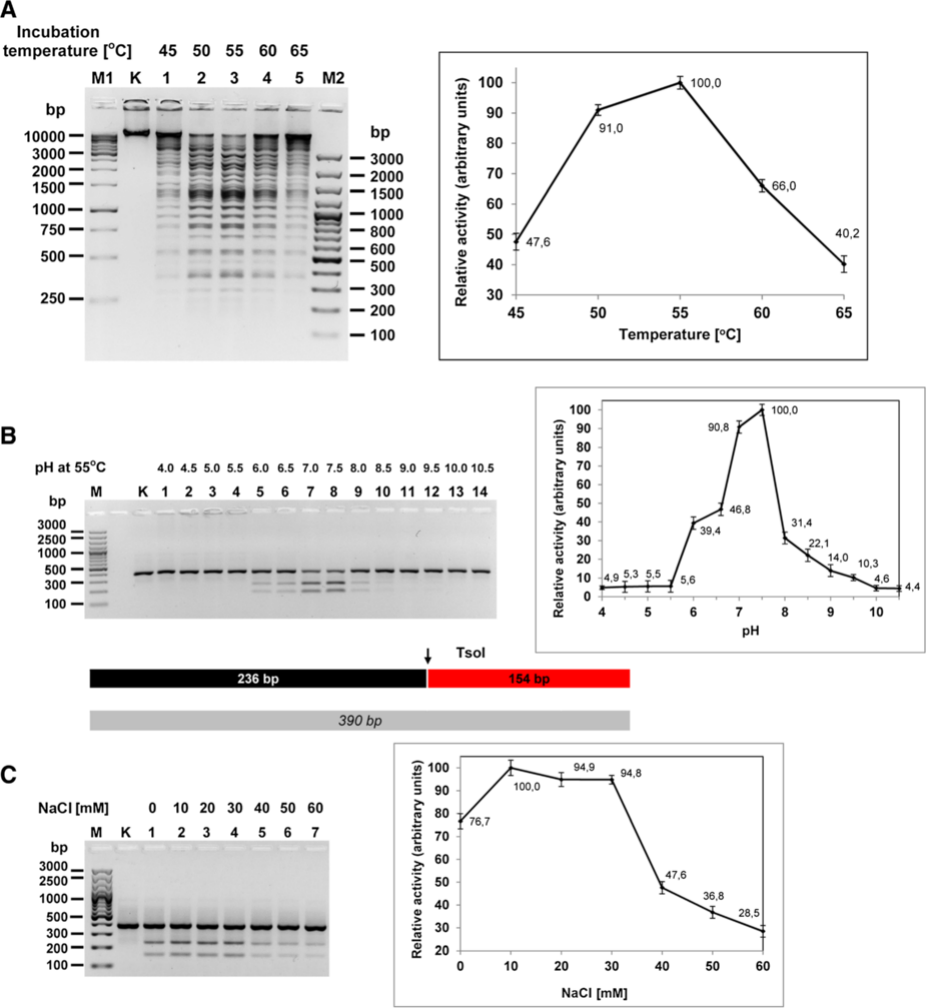
**Evaluation of temperature, pH and salt concentration effect on TsoI REase activity. (A)** The optimum temperature range of TsoI REase. 0.5 μg of T7 DNA (= 0.02 pmol recognition sites) was digested with 1 pmol (0.05 u) of TsoI in standard buffer supplemented with 50 μM SAM for 30 min in the temperature range from 45 to 65°C. Lane M1, GeneRuler™ 1 kb DNA Ladder (Thermo Fisher Scientific (Fermentas); selected bands marked); lane K, undigested T7 DNA; lane 1, 45°C; lane 2, 50°C; lane 3, 55°C; lane 4, 60°C; lane 5, 65°C; lane M2, GeneRuler™ 100 bp Plus DNA Ladder (Fermentas), selected bands marked. **(B)** The pH activity range of TsoI REase. 0.3 μg (= 0.6 pmol recognition sites) single site PCR substrate (390 bp) was digested with 0.33 pmol TsoI (0.016 u) in the pH range from 4.0 to 10.5 for 30 min at 55°C as described in the Methods section. GeneRuler™ 100 bp Plus DNA Ladder (Thermo Fisher Scientific (Fermentas); selected bands marked); lane K, undigested PCR fragment; lanes 1–14, PCR fragments after incubation with wt TsoI at indicated pH values: lane 1, at pH 4.0; lane 2, 4.5; lane 3, 5.0; lane 4, 5.5; lane 5, 6.0; lane 6, 6.5; lane 7, pH 7.0; lane 8, 7.5; lane 9, 8.0; lane 10, 8.5; lane 11, 9.0; lane 12, 9.5; lane 13, 10.0; lane 14, 10.5. **(C)** The influence of ionic strength on TsoI REase activity. 0.3 μg (= 0.6 pmol recognition sites) single site PCR substrate (390 bp) was digested with 0.33 pmol TsoI (0.016 u) in the NaCl concentration range from 0 to 60 mM for 30 min at 55°C in the standard reaction buffer, devoid of initial NaCl content: 10 mM Tris–HCl, pH 7.5, 10 mM MgCl_2_, 0.01 mg/ml BSA, 0.5 mM DTT, 50 μM SAM. Lane M, GeneRuler^TM^ 100 bp Plus DNA Ladder (Thermo Fisher Scientific (Fermentas); selected bands marked); lane K, undigested PCR fragment; lanes 1–7, digested PCR fragment: lane 1, without NaCl; lane 2, with 10 mM; lane 3, 20 mM; lane 4, 30 mM; lane 5, 40 mM; lane 6, 50 mM; lane 7, 60 mM.

Further evaluation of wt TsoI properties included molecular sieving in the reaction buffer ‘G’ (without SAM and BSA) (not shown) [4]. The experiment showed that the native REase elutes as a monomer, just like other *Thermus* sp. family enzymes, confirming their common organisation scheme [3-8]. Activity temperature profiling has shown somewhat surprising results, indicating that TsoI is the least thermostable enzyme in the *Thermus* sp. family, with an optimum at 55°C and retaining only 18.4% activity at 65°C (Figure 9A). We previously showed that the typical optimum temperature for *Thermus* sp. enzymes is 65-75°C [3-8]. The reaction optimum of TsoI is considerably lower, by app. 10-15°C, even though its natural host *T. scotoductus* grows optimally at 67°C [2] (Lubys Arvydas, personal communication). This indicates that certain cellular components may be much more sensitive than an organism as a whole entity and are still able to fulfil their function. Also, such a property may be reminiscent of a past acquisition of TsoI coding genes by *T. scotoductus* from less thermophilic bacteria. The pH influence on the TsoI REase activity was determined. Trace REase activity was detected in the 4.0-5.5 range; while activity increased from 95.5 to 100% in the 7.0-7.5 range, and decreased from 20.8 to 7.8% in the 8.0-8.5 range (Figure 9B). The results obtained were similar to those described for other *Thermus* sp. family members. At optimised pH (=7.5), a buffer with variable concentrations of NaCl was used to determine optimal ionic strength. The maximum activity was close to 100% in the relatively wide 10–30 mM concentration range, and decreased gradually to 60 mM NaCl (Figure 9C). For practical applications of TsoI in DNA manipulations, 50 mM NaCl is preferred, as a compromise between maximum enzymatic activity versus enzyme stability during the reaction and lowered “star” activity.

## Conclusions

i. The prototype TsoI REase gene was cloned in *E. coli* and sequenced using a new modification of a classic biochemical selection method, where the positive selection vector was combined with two rounds of selection for the methylation phenotype.

ii. Expression of a cloned *tsoIRM* gene under a T7 promoter, yielding enzymatically active bifunctional TsoI REase-MTase, required an additional round of the biochemical selection of expression subclones to eliminate abundant spontaneous mutants.

iii. TsoI is a member of the *Thermus* sp. modular enzyme family and the TspDTI-subfamily, exhibiting a rare phenomenon among REases – relatively high homologies to TspDTI, Tth111II and TthHB27I, even though they recognise distinct cognate sites.

iv. Within the recombinant TsoI bifunctional enzyme, MTase dominates REase activity both *in vivo* and *in vitro* in crude lysates assays. This may suggest the existence of an additional biological role different than the restriction of invading DNA.

v. Reaction parameters and cofactor requirements were determined, including a surprisingly low temperature optimum of 55°C and lower than expected *tsoIRM* ORF GC content, which suggests the occurrence of horizontal gene transfer in the past.

## Methods

### Bacterial strains, plasmids, media and reagents

The TsoI-producing strain *Thermus scotoductus* RFL4 was obtained from the Thermo Fisher Scientific Fermentas (Vilnius, Lithuania) collection and cultivated at 67°C in modified Luria broth either in flasks or in a fermenter (details on fermentation and detailed composition of broth are available under request). *E. coli* ER2566 {*fhuA2 lacZ::T7 gene1 [lon] ompT gal sulA11 R(mcr-73::miniTn10--*TetS*)2 [dcm] R(zgb-210::Tn10--*TetS*) endA1* Δ*(mcrC-mrr)114::IS10*} (New England Biolabs, MA, USA) were used for all cloning and expression procedures. *E. coli* bacteria were grown in LB medium [20]. Media were supplemented with 50 mg/mL kanamycin and 50 mg/mL ampicillin for pSEKm’-MCS vector, and 50 mg/mL ampicillin for pET21NS vector. Difco media were from Becton- Dickinson (Franklin Lakes, NJ, USA), DNA ladders and protein size standard, DNA purification kits, restriction enzymes, λ DNA, T4 DNA polymerase, T4 DNA ligase, Taq DNA polymerase, alkaline phosphatase and PCR primers were from Thermo Fisher Scientific/Fermentas (Vilnius, Lithuania). T7 DNA was from Vivantis Technologies Sdn. Bhd. (Malaysia). The expression vector pET21NS (Fermentas) was a modification of the pET21 vector (Novagen, WI, USA) (AmpR, MCS, *col* E1 *ori*, f1 *ori*, and T7-lac promoter), containing NotI and SmiI restriction sites introduced into MCS. All other reagents were purchased from Sigma-Aldrich (St. Louis, MO, USA). Sequencing was carried out using the ABI Prism 310 automated sequencer with the ABI Prism BigDye Terminator Cycle Sequencing Ready Reaction Kit (Perkin Elmer Applied Biosystems, Foster City, CA, USA). The sequence data were analysed using ABI Chromas 1.45 software (Perkin Elmer Applied Biosystems) and either Vector NTI (Invitrogen, CA, USA) or DNAIS MAX /DNASIS 2.5 software (Hitachi Software, San Bruno, CA, USA).

### Native (wt) and recombinant TsoI sources

The native (wt) TsoI enzyme was first found and purified to homogeneity from *T. scotoductus*. All purification steps were carried out at +4°C. Frozen biomass of *T. scotoductus* was thawed in buffer A (10 mM K-phosphate, 1 mM EDTA, 7 mM 2-mercaptoethanol, pH 7.4) containing 0.1 M KCl. Cells were disrupted by sonication. Following the sonication, insoluble material was removed by centrifugation. The supernatant was applied to a phosphocellulose P11 column pre-equilibrated with buffer A plus 0.1 M KCl. The column was washed with the same buffer, and elution of bound enzymes was performed using the buffer A and the linear gradient of KCl from 0.1 to 0.8 M. Individual fractions were tested for the REase activity. The TsoI enzyme was eluted at between 0.4 and 0.6 M KCl. The peak fractions were pooled, dialyzed against 20 volumes of buffer A supplemented with 0.2 M NaCl, and applied to a Blue Sepharose column. After the washing of the column with the A buffer (+ 0.2 M NaCl) the elution of bound enzymes was accomplished using 0.2–1.0 M gradient of NaCl in buffer A. The REase activity was eluted from the column between 0.6 and 0.8 M NaCl. The peak fractions were pooled, dialysed against 20 volumes of buffer A supplemented with 0.2 M KCl, and the TsoI pool from Blue Sepharose was then fractionated on a Heparin Sepharose column pre-equilibrated with buffer A plus 0.2 M KCl. The column was washed with the same buffer and the enzyme was eluted using a linear gradient between 0.2 to 0.9 M KCl in buffer A. REase activity was found in fractions eluted at 0.40-0.60 M KCl. These fractions were pooled, supplemented with BSA (final concentration 0.05 mg/ml) and dialysed against 10 volumes of 10 mM Tris–HCl (pH 7.4), 100 mM KCl, 1 mM EDTA, 1 mM DTT and 50% glycerol. The final preparation was stored at −20°C.

Recombinant TsoI was prepared for activity testing by sonication-mediated disruption of *E. coli* cells followed by cell debris centrifugation. Since the TsoI protein expression in plasmid constructs was at a high level, where the corresponding TsoI band in the final expression construct dominated other proteins present in the cleared lysates, such a partial purification procedure was sufficient to conduct the experiments described.

### Cloning and determination of the nucleotide sequence of the *tsoIRM* gene

The *tsoIRM* gene and its flanking regions were cloned using a positive selection vector combined with a two-stage biochemical selection procedure [15] of the library prepared from *T. scotoductus* genomic DNA. [15]. The genomic DNA was isolated as described [20] and was subjected to limited random sharing with the use of ultrasound.

The fragmentation was monitored by agarose gel electrophoresis to identify the conditions favourable for obtaining DNA fragments larger than 3 kb (the expected size of the *tsoIRM* gene based on the size of wt TsoI). The fragments were used for library construction in the pSEKm’-MCS vector. The vector was linearized with Eco32I having a unique target within the positive selection gene *eco47IR* which codes for the restriction endonuclease Eco32I, and dephosphorylated. Genomic fragments were T4 DNA Polymerase/dNTPs blunted [20] and ligated with a Eco32I-linearised vector, using an app. 3: 1 molar ratio of insert: vector molecules (assuming an average genomic fragment length of app. 5 kb). The ligation mixture was used to electroporate competent *E. coli* ER2566 cells and transformants were plated onto LB/kanamycin+ampicillin plates. From the obtained library of 8 × 10^5^ clones recombinant plasmids were isolated *in toto* from the pooled library and were subjected to two consecutive rounds of biochemical selection. The plasmid pool (1 μg) was digested overnight with an excess (5 units) of native, *T. scotoductus* -isolated TsoI REase; the reaction mixture was purified using phenol/chloroform extraction [20], DNA products precipitated with the ethanol, dissolved in 5 mM Tris–HCl pH 8.0 and transformed by electroporation back to the same ER2566 strain. The resulting 2800 colonies were pooled again, the plasmids were isolated *in toto* and were subjected again to TsoI biochemical selection. Fifty colonies out of 120 obtained after the second round of biochemical selection/transformation were subjected to individual analysis with colony PCR, used for screening (using the vector’s flanking standard primers) for plasmids carrying fragments larger than 3 kb, and 14 plasmids among 50 analysed were found to fulfil this criterion. Selected plasmids were subjected to HindIII digestion to locate the same insert-derived DNA fragments, which would indicate that they originated from the same genomic region. Restriction mapping revealed seven types of plasmids all possessing the same cloned HindIII fragments of 1.2 and 1.5 kb in size. Of these, plasmids representing five smallest isolates were used for both methylation analysis and sequencing of cloned regions. The combination of both strand sequences resulted in a 4365 bp genomic DNA segment, where TsoI ORF was detected.

### Overexpression of the *tsoIRM* gene under T7-lac promoter in *E. coli*

A specially designed pET21 derivative pET21NS was used for the directional cloning of the NotI-SmiI cleaved PCR fragment encompassing the full-length *tsoIRM* gene. The 15 resulting recombinant plasmids were introduced into *E. coli* ER2566 for expression trials. Recombinant strains were grown overnight at 37°C in 5 ml LB, supplemented with ampicillin. 100 μl of the overnight culture were used to inoculate 5 ml of ampicillin-supplemented fresh LB, grown at 37°C until OD600 = 0.5-0.8, induced with 1 mM IPTG and allowed to grow further at 37°C for 3 hours. Crude cell extracts exhibited only trace the amounts of TsoI REase activity, while SDS-PAGE demonstrated abundant amounts of a large enzyme that appeared after induction. Testing of the TsoI protection level of all 15 plasmids before and after induction showed that all were unprotected before induction and only partially protected after induction. Thus, considering the possibility of the appearance and selection for PCR errors in the large, toxic *tsoIRM* gene, the few expression constructs were subjected to DNA sequencing, which revealed the presence of multiple mutations. To select for mutation-free plasmids, 5000 ampicillin-resistant colonies obtained after the transformation of ER5266 by (pET21NS + *tsoIRM*-PCR fragment) ligation mixture were pooled and used to inoculate 100 ml LB supplemented with ampicillin. Cells were grown at 37°C until OD_600_ = 0.7, TsoI expression was induced by the addition of 1 mM IPTG. The culture was further grown at 37°C for 4 hours and then used for the isolation of total plasmid DNA. The latter has 7 TsoI targets and thus can be enriched for more active MTase variants using the same biochemical selection approach. Total plasmid DNA was cleaved with TsoI and the reaction mixture transformed back to *E. coli* ER2566 (3^rd^ round of biochemical selection). The 20 individual colonies resulting from the transformation were again tested for TsoI activity in crude cell extracts and for the TsoI protection level of plasmids carrying the *tsoIRM* gene, isolated from induced cultures. Crude cell extracts exhibited much higher TsoI activity, while expression plasmids of producing strains were almost completely protected from TsoI action when isolated after induction. Four of these plasmids were sequenced, and two were found to have no mutations in the PCR-amplified TsoI-coding gene. One of these two plasmids was called pET21NS-TsoIRM and was used for further experiments.

### REase and MTase assays

#### REase assays

For REase assays various modifications of standard TsoI reaction conditions were used, which provide a compromise between enzyme stability, lowest “star” activity and DNA cleavage efficiency. Typically, reactions were performed in 50 μl of the reaction buffer, containing 10 mM Tris–HCl pH 7.5 at 37°C, 10 mM MgCl_2_, 50 mM NaCl, 0.5 mM DTT, 0.1 mg/ml BSA and supplemented with 50 μM SAM. The following DNAs were used as test substrates: λDNA, T7 bacteriophage DNA, PCR fragment with a single TsoI site and DNA of recombinant plasmids (for biochemical selection and protection assays). One unit of TsoI REase is defined as the amount of enzyme required to hydrolyse 1 μg of λ DNA in an hour at 55°C in 50 μl of standard TsoI buffer, enriched with 50 μM SAM, resulting in a stable partial DNA cleavage pattern.

Quantitative evaluations of temperature, pH, salt concentrations were determined using DNA cleavage reactions under enzyme-limiting conditions. Comparative densitometry was performed on selected reference DNA bands from photographs of ethidium bromide and/or Sybr Green stained gels, taken under various exposure times. The temperature reaction optimum was determined in standard TsoI buffer. The NaCl concentration optimum was determined in standard TsoI buffer, devoid of initial salt addition. The pH reaction optimum was evaluated in three buffer systems, each dedicated to the maximum buffering capacity range: sodium acetate-acetic acid of pH from 4.0 to 5.5, HEPES-KOH from 6.0 to 7.0, and Tris–HCl buffer from 7.5-10.5. The pH of the reaction buffers was adjusted at 55°C after all the buffer components had been dissolved. The cleavage reactions were performed under enzyme-limiting conditions, (0.55: 1 molar ratio of the enzyme to cognate sites for 30 min).

#### Methyltransferase assays

The *in vitro* methylation activity of the TsoI enzyme was tested by the DNA protection assay, in 50 μl of TsoI standard buffer (without Mg^2+^) supplemented with 50 μM SAM. After the addition of recombinant TsoI protein present in cleared lysates, the reaction mixtures were incubated at 55°C. The cleavage products visible after the incubation with crude cell extract, resulted from the resident TsoI REase activity in the presence of Mg^2+^ ions (Figure 7, lanes 9,12). Samples were purified to remove TsoI protein by chloroform extraction and then DNA precipitated with isopropanol. Modified DNA was challenged with an excess of wt TsoI (2 units, app. 2: 1 M ratio of enzyme to recognition sites) for an hour in 50 μl of standard TsoI buffer supplemented with 50 μM SAM at 55°C. The reaction products were then resolved by agarose gel electrophoresis.

## Competing interests

Arvydas Lubys, Jolanta Vitkute, Goda Mitkaite are affiliated with Thermo Fisher Scientific Inc. (USA), Fermentas branch (Vilnius, Lithuania) and provided scientific information concerning *tsoIRM* gene cloning, TsoI amino acid sequence and selected enzyme features. The authors declare that they have no competing interest.

## Authors’ contributions

AL conceived and coordinated the TsoI cloning project. PS coordinated the native TsoI characterisation experiments, came up with the concept of the new *Thermus* sp. enzyme family and drafted the manuscript. DR and GM performed the TsoI gene cloning and preliminary expression experiments. JZ and JF participated in the enzyme characterisation experiments. AZS performed some enzyme characterisation experiments, participated in the design and interpretation of the experimental analyses, prepared all figures and drafted the manuscript. All authors read and approved the final manuscript.
